# Guiding Therapy by Coronary CT Angiography Improves Outcomes in Patients With Stable Chest Pain

**DOI:** 10.1016/j.jacc.2019.07.085

**Published:** 2019-10-22

**Authors:** Philip D. Adamson, Michelle C. Williams, Marc R. Dweck, Nicholas L. Mills, Nicholas A. Boon, Marwa Daghem, Rong Bing, Alastair J. Moss, Kenneth Mangion, Marcus Flather, John Forbes, Amanda Hunter, John Norrie, Anoop S.V. Shah, Adam D. Timmis, Edwin J.R. van Beek, Amir A. Ahmadi, Jonathon Leipsic, Jagat Narula, David E. Newby, Giles Roditi, David A. McAllister, Colin Berry, SCOT-HEART Investigators

**Affiliations:** a British Heart Foundation Centre for Cardiovascular Science, University of Edinburgh, Edinburgh, United Kingdom; b Christchurch Heart Institute, University of Otago, Christchurch, New Zealand; c Edinburgh Imaging, Queen’s Medical Research Institute University of Edinburgh, Edinburgh, United Kingdom; d Institute of Cardiovascular and Medical Sciences, University of Glasgow, Glasgow, United Kingdom; e Norwich Medical School, University of East Anglia, Norwich, United Kingdom; f Health Research Institute, University of Limerick, Limerick, Ireland; g Edinburgh Clinical Trials Unit, University of Edinburgh, Edinburgh, United Kingdom; h William Harvey Research Institute, Queen Mary University of London, London, United Kingdom; i Ichan School of Medicine and Mount Sinai Hospital, Mount Sinai Heart, New York, New York; j St. Paul’s Hospital, University of British Columbia, Vancouver, British Columbia, Canada; k Institute of Health and Wellbeing, University of Glasgow, Glasgow, United Kingdom

**Keywords:** angina pectoris, computed tomography, coronary heart disease, CI, confidence interval, CTA, computed tomography angiography, HR, hazard ratio, NHS, National Health Service

## Abstract

**Background:**

Within the SCOT-HEART (Scottish COmputed Tomography of the HEART Trial) trial of patients with stable chest pain, the use of coronary computed tomography angiography (CTA) reduced the rate of death from coronary heart disease or nonfatal myocardial infarction (primary endpoint).

**Objectives:**

This study sought to assess the consistency and mechanisms of the 5-year reduction in this endpoint.

**Methods:**

In this open-label trial, 4,146 participants were randomized to standard care alone or standard care plus coronary CTA. This study explored the primary endpoint by symptoms, diagnosis, coronary revascularizations, and preventative therapies.

**Results:**

Event reductions were consistent across symptom and risk categories (p = NS for interactions). In patients who were not diagnosed with angina due to coronary heart disease, coronary CTA was associated with a lower primary endpoint incidence rate (0.23; 95% confidence interval [CI]: 0.13 to 0.35 vs. 0.59; 95% CI: 0.42 to 0.80 per 100 patient-years; p < 0.001). In those who had undergone coronary CTA, rates of coronary revascularization were higher in the first year (hazard ratio [HR]: 1.21; 95% CI: 1.01 to 1.46; p = 0.042) but lower beyond 1 year (HR: 0.59; 95% CI: 0.38 to 0.90; p = 0.015). Patients assigned to coronary CTA had higher rates of preventative therapies throughout follow-up (p < 0.001 for all), with rates highest in those with CT-defined coronary artery disease. Modeling studies demonstrated the plausibility of the observed effect size.

**Conclusions:**

The beneficial effect of coronary CTA on outcomes is consistent across subgroups with plausible underlying mechanisms. Coronary CTA improves coronary heart disease outcomes by enabling better targeting of preventative treatments to those with coronary artery disease. (Scottish COmputed Tomography of the HEART Trial [SCOT-HEART]; NCT01149590)

Coronary computed tomography angiography (CTA) has high sensitivity and specificity for the detection of coronary artery disease 1, 2. This has prompted the evaluation of coronary CTA as a diagnostic test for coronary artery disease in patients presenting with stable chest pain. The short-term benefits of coronary CTA in this population have included better diagnostic certainty, lower rates of normal coronary arteries at the time of invasive coronary angiography, and improved targeting of symptomatic and preventative therapies 3, 4. Large-scale clinical trials have also suggested that short-term coronary heart disease events are reduced 4, 5. In the SCOT-HEART (Scottish COmputed Tomography of the HEART Trial), we recently reported the effects of coronary CTA on the pre-specified 5-year clinical outcomes including investigations, treatments, and clinical events (6). We demonstrated that an initial strategy of coronary CTA was associated with a 41% relative risk reduction in coronary heart disease death or nonfatal myocardial infarction at 5 years. This major reduction in events has prompted questions about the mechanisms of benefit, the potential for bias, and the plausibility of the effect size.

We here present further analyses of the 5-year data from the SCOT-HEART trial to assess the robustness of the event reductions seen with coronary CTA with respect to the participant subgroups, the changes in diagnosis, and the alterations to procedural and pharmacological treatments. Drawing these disparate effects together, we wanted to determine the overall attribution of benefits in relation to the primary endpoint and the study intervention effect size.

## Methods

The study population and trial design were reported previously 4, 5, 7. In brief, adult patients age ≤75 years who attended the outpatient cardiology clinic with stable chest pain were invited to participate in the trial in 12 cardiology centers across Scotland between 2010 and 2014. They were randomized 1:1 to standard care alone or standard care plus coronary CTA. Attending clinician-directed changes in diagnosis, investigations, and treatments were documented at 6 weeks, and changes in investigations, treatments, and clinical outcomes over 5 years were obtained from nationwide routinely collected health care data through National Health Service (NHS) Scotland.

### Symptoms

Participants without a prior history of coronary heart disease comprised 91% of the study population and were categorized into those with nonanginal chest pain and a normal 12-lead electrocardiogram (nonanginal chest pain), and those with either typical or atypical chest pain, or nonanginal chest pain and an abnormal electrocardiogram (possible angina) as per the National Institute for Health and Care Excellence guidelines 7, 8.

### 10-year cardiovascular risk

The 10-year cardiovascular risk of participants was determined by the ASSIGN score. This score is based on traditional cardiovascular risk factors but also incorporates other risk markers including social deprivation and family history of cardiovascular disease and has been calibrated for the Scottish population (9). To avoid potential for bias, changes in diagnosis, investigations, and treatments were documented following prompting with either the coronary CTA result (standard care and coronary CTA) or the ASSIGN score (standard care alone) (10).

### Coronary revascularization

Coronary revascularization procedures (percutaneous coronary intervention and coronary artery bypass graft surgery) were identified from inpatient and day-case episodes, and coronary disease burden was quantified using centralized review of individual coronary angiograms blinded to allocated intervention 3, 4, 6.

### Preventative therapies

The Scottish national community drug-prescribing database of the Information and Statistics Division in NHS Scotland maintains a detailed record of all NHS prescriptions dispensed in the community, which are linked to individual patient identifiers 3, 4, 6. All prescriptions are dispensed by community pharmacies, dispensing doctors, and a small number of specialist suppliers.

### Clinical follow-up

Clinical data and deaths were obtained from the Information and Statistics Division and the electronic Data Research and Innovation Service of NHS Scotland up to March 31, 2018. To account for reporting delays in data completeness (6 to 8 weeks), outcomes were determined up to January 31, 2018. Previous analyses have demonstrated an excellent correlation (>95%) between clinical events identified through Scottish health record linkage and clinical events recorded and adjudicated as part of a regulated clinical trial (11). This has been successfully applied in other settings 12, 13 and for longer-term clinical trial follow-up (14).

### Statistical analysis

This was a post hoc analysis of the SCOT-HEART trial. Clinical outcomes were analyzed using Cox regression, adjusted for center and minimization variables, with cumulative event curves constructed. We performed a landmark analysis at 12 months, reasoning that coronary CTA–driven alterations in invasive coronary angiography and coronary revascularization should have been completed by this time point. Data are presented as mean ± SD, median (interquartile range), and hazard ratio (HR) or odds ratio (95% confidence interval [CI]) as appropriate. No correction for multiplicity was undertaken when testing secondary or other outcomes.

As the SCOT-HEART trial randomized the application of coronary CTA as a diagnostic intervention, it cannot definitively determine the mediation pathway for the treatment effects demonstrated. To explore the plausibility of the treatment effect, we performed a modeling study aiming to estimate the mean outcome for patients in the coronary CTA group under a counterfactual scenario wherein the management of patients in the coronary CTA group was the same as that observed in the standard care group. The full set of assumptions are given in the Online Appendix. However, we wish to highlight that we modeled a scenario wherein the highest risk (most appropriate) patients were those who had received additional preventative therapies as a result of the coronary CTA intervention given that this would be the consequence of improved diagnostic precision. Under the observed difference between the trial arms in use of preventative therapies, this represents a best-case scenario.

Statistical significance was taken as a 2-sided p < 0.05. All analysis was undertaken using R version 3.5.0 (R Foundation, Vienna, Austria).

## Results

We randomized 4,146 patients with stable chest pain at 12 cardiology centers across Scotland to either standard of care alone (n = 2,073) or standard of care plus coronary CTA (n = 2,073). Participants were middle aged, had a high prevalence of cardiovascular risk factors, and had a slight male preponderance (Table 1).

**Table 1:** Baseline Characteristics of Trial Participants

	All Participants (N = 4,146, 100%)	Standard Care + Coronary CTA (n = 2,073, 50%)	Standard Care (n = 2,073, 50%)
Male	2,325 (56)	1,162 (56)	1,163 (56)
Age, yrs	57 ± 10	57 ± 10	57 ± 10
Chest pain group			
Nonanginal chest pain	1,447 (35)	712 (34)	735 (35)
Possible angina	2,323 (56)	1,174 (57)	1,149 (56)
Prior CHD	372 (9)	186 (9)	186 (9)
Risk factors			
Smoking habit	2,185 (53)	1,095 (53)	1,090 (53)
Hypertension	1,395 (34)	712 (34)	683 (33)
Diabetes mellitus	444 (11)	223 (11)	221 (11)
Hypercholesterolemia	2,176 (53)	1,099 (53)	1,077 (52)
Family history of CHD	1,716 (41)	887 (43)	829 (40)
Baseline therapy			
Antiplatelet agent	1,993 (48)	1,009 (49)	984 (48)
Statin	1,786 (43)	902 (44)	884 (43)
Predicted 10-yr CHD risk, %	17 ± 12	18 ± 11	17 ± 12

Values are n (%) or mean ± SD.

CHD = coronary heart disease; CTA = computed tomography angiography.

### Symptoms and prior coronary heart disease

**Figure 1:**
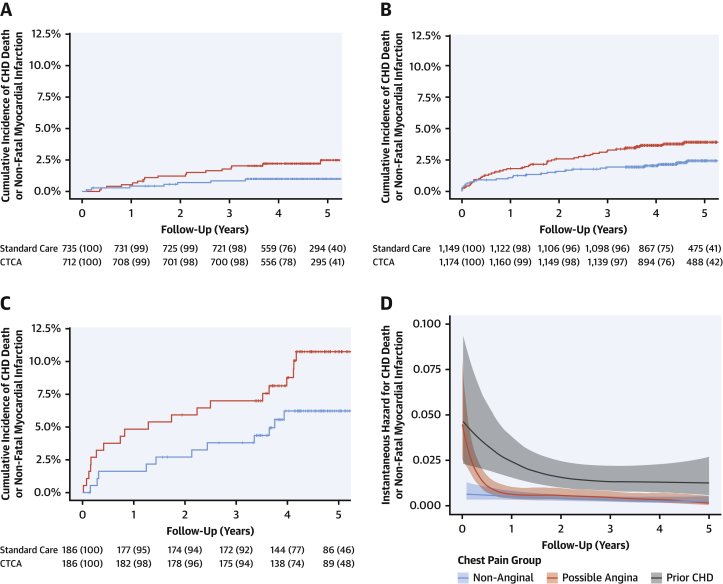
Cumulative Incidence of CHD Death or Nonfatal MI Cumulative incidence curves for coronary heart disease (CHD) death or nonfatal myocardial infarction (MI) in **(A)** patients with nonanginal chest pain, **(B)** patients with possible angina, and **(C)** patients with prior CHD, allocated to standard care alone **(red)** and computed tomography coronary angiography (CTCA) plus standard of care **(blue). (D)** Instantaneous hazards over time for each of the 3 chest pain groups. Patients in the nonanginal group **(blue)** have a low risk of the primary endpoint that is constant over time. Patients in the prior CHD group **(gray)** are at highest risk but the magnitude of risk is greatest during the first 1 to 2 years. Patients in the possible angina group **(red)** have a high early risk that rapidly declines over the first 6 to 12 months.

Of the 4,146 study participants, there were 1,447 patients with nonanginal chest pain and a normal resting electrocardiogram, 2,323 with possible angina, and 372 with prior coronary heart disease. Four patients were excluded due to incomplete symptom descriptions. Nonobstructive or obstructive disease was identified on coronary CTA in 49.9% of those with nonanginal chest pain and 66.9% of those with possible angina (Table 2). All 3 groups derived similar relative benefit from coronary CTA (p value for interaction ≥0.50), although the absolute magnitude and temporal pattern of benefit appeared to vary (Figure 1). Among patients with nonanginal chest pain, the event rate appeared relatively constant over time with the benefits of coronary CTA only apparent over a more prolonged time period (>1 year). In contrast, patients with prior coronary heart disease were at higher risk of events, especially in the early follow-up period, and the benefits of coronary CTA were more immediately apparent. Compared with these groups, patients with possible angina were at intermediate risk and demonstrated an intermediate time course of benefit.

**Table 2:** Coronary CTA Findings According to Diagnostic Classification as Defined by the NICE Guideline for the Assessment of Chest Pain

	Coronary CTA Result		
Diagnostic classification	Normal	Nonobstructive	Obstructive
Nonanginal (n = 591)	296 (50.1)	239 (40.4)	56 (9.5)
Possible angina (n = 1,028)	340 (33.1)	385 (37.5)	303 (29.5)
Prior CHD (n = 162)	13 (8.0)	56 (34.6)	93 (57.4)

Values are n (%) and include only those with a diagnostic coronary CTA result available.

NICE = National Institute of Health and Care Excellence; other abbreviations as in Table 1.

### Diagnosis

**Figure 2:**
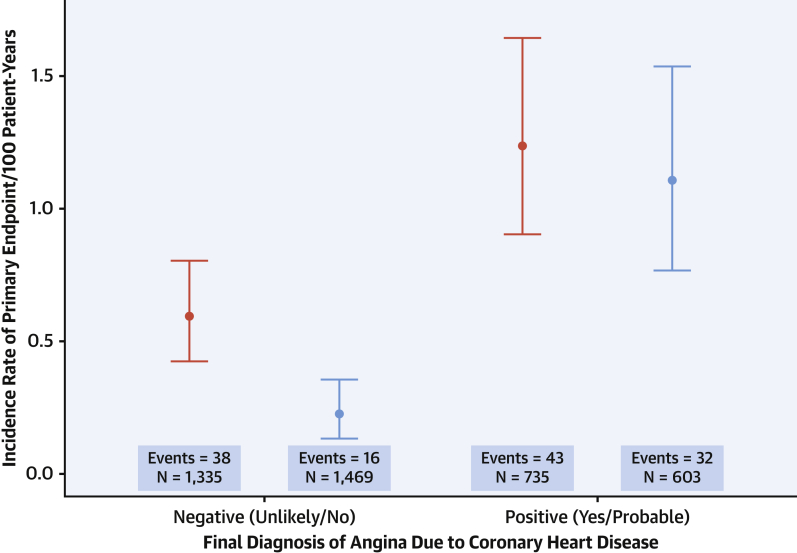
5-Year Incidence Rates of CHD Death or Nonfatal MI Five-year incidence rates of CHD death or nonfatal MI in patients with **(right)** and without **(left)** a diagnosis of angina due to CHD 6 weeks after randomization according to the trial allocation of standard care alone **(red)** and computed tomography coronary angiography plus standard of care **(blue).** Abbreviations as in Figure 1.

We have previously reported that the diagnosis of angina due to coronary heart disease was changed in 1 in 4 patients following coronary CTA (4). Among patients where the diagnosis of angina due to coronary heart disease was not made, those in the coronary CTA group had a lower incidence rate of coronary heart disease death or nonfatal myocardial infarction compared with the standard care group (incidence rate: 0.23; 95% CI: 0.13 to 0.35 vs. 0.59; 95% CI: 0.42 to 0.80 per 100 patient-years, respectively; p < 0.001) (Figure 2). The findings on coronary CTA were strongly predictive of future risk of coronary heart disease death or nonfatal myocardial infarction (Online Figure 1).

### Coronary angiography and revascularization

**Figure 3:**
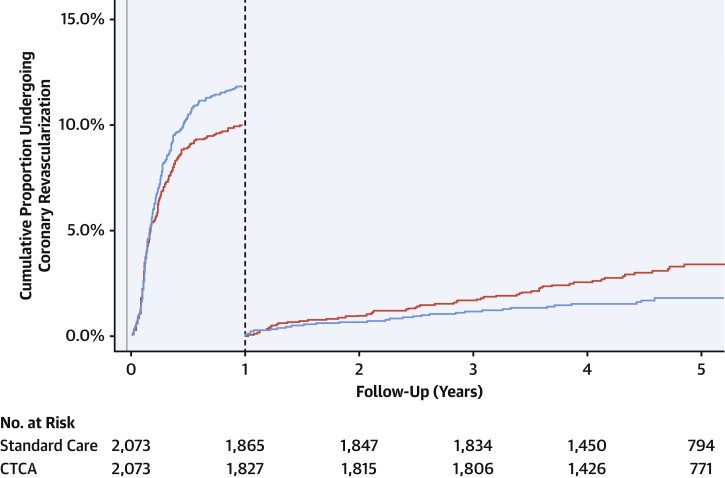
Cumulative Incidence of Coronary Revascularization Within the First Year and Beyond 1 Year Landmark analysis demonstration cumulative incidence curves for coronary revascularization within the first year and beyond 1 year in patients allocated to standard care alone **(red)** and computed tomography coronary angiography (CTCA) plus standard of care **(blue).**

Among patients referred for invasive coronary angiography, those randomized to CTA had more extensive burden of coronary artery disease (p = 0.056) (Table 3). This finding was particularly notable among participants without inducible ischemia on exercise testing (Online Table 1). At the end of 1 year, more patients who had undergone coronary CTA received coronary revascularization (246 vs. 208; HR: 1.21; 95% CI: 1.01 to 1.46; p = 0.042) (Figure 3). Coronary revascularization was predominantly percutaneous coronary intervention (n = 353; 8.5%) although one-quarter of patients (n = 111; 2.7%) underwent coronary artery bypass graft surgery. Beyond 1 year, 33 coronary revascularizations occurred in the coronary CTA group compared with 59 in the standard care group (HR: 0.59; 95% CI: 0.38 to 0.90; p = 0.015). Of these, 8 coronary revascularizations were prompted by myocardial infarction in the coronary CTA group, and 18 in the standard care group (HR: 0.45; 95% CI: 0.20 to 1.04; p = 0.061).

**Table 3:** Findings on Invasive Coronary Angiography Performed Within 1 Year of Randomization

	Standard Care	Coronary CTA	p Value
Number of coronary arteries with ≥50% stenosis			0.014∗
0	157 (39.3)	120 (28.6)	
1	109 (27.3)	135 (32.3)	
2	73 (18.3)	93 (22.2)	
3+	60 (15.0)	71 (16.9)	
Number of coronary arteries with ≥70% stenosis†			0.059∗
0	176 (44.1)	152 (36.3)	
1	125 (31.3)	144 (34.4)	
2	54 (13.5)	74 (17.7)	
3+	44 (11.0)	49 (11.7)	
Prognostically important CAD‡	76 (19.0)	94 (22.4)	0.268§

Values are n (%).

CAD = coronary artery disease; CTA = computed tomography angiography.

∗ These p values were determined from Cochran-Armitage test for trend.

† Also includes ≥50% stenosis of left main coronary artery.

‡ Prognostically important CAD defined as any of the following: ≥50% stenosis of left main coronary artery; ≥70% stenosis of at least 3 main epicardial arteries; or ≥70% stenosis of at least 2 epicardial arteries including the proximal left anterior descending artery.

§ This p value was determined using Pearson chi-square test.

### Preventative therapies

**Figure 4:**
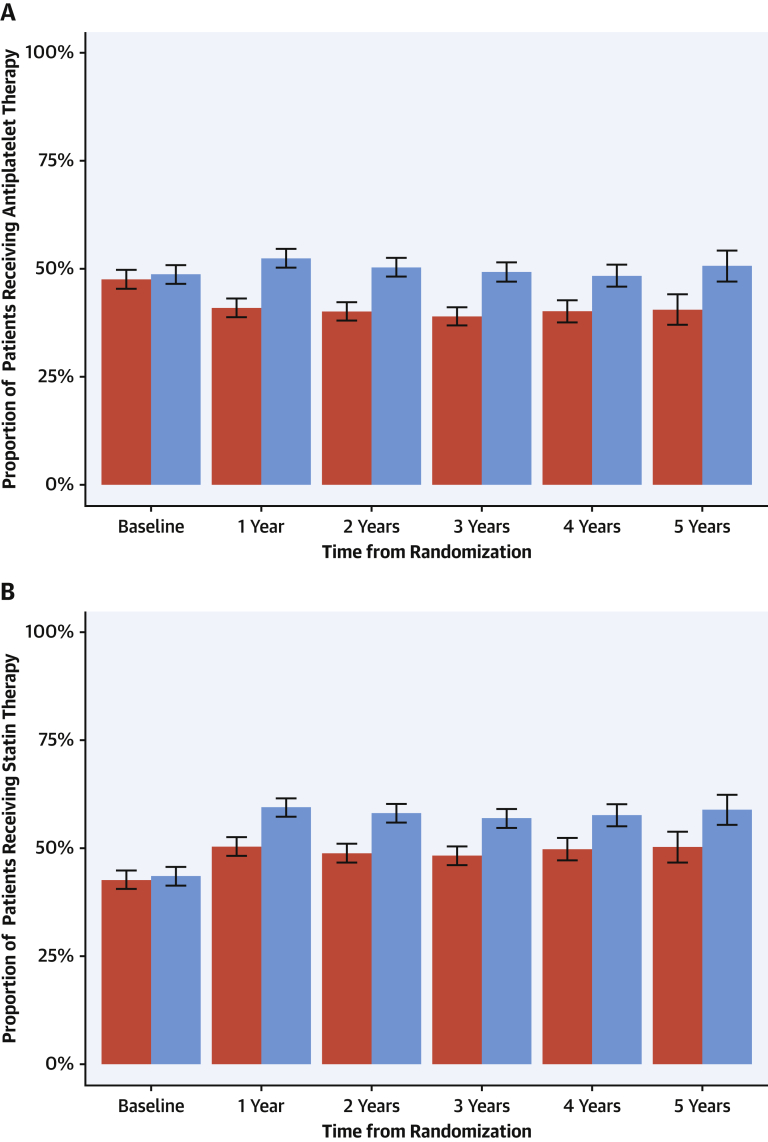
Prescribing of Preventative Therapy Over 5 Years of Follow-Up Frequency of prescribing for **(A)** antiplatelet and **(B)** statin therapy across 5 years in patients allocated to standard care alone **(red)** and computed tomography coronary angiography plus standard of care **(blue).** The **error bars** relate to confidence intervals for comparison between trial arms. p < 0.001 for all comparisons except baseline where p = NS.

**Figure 5:**
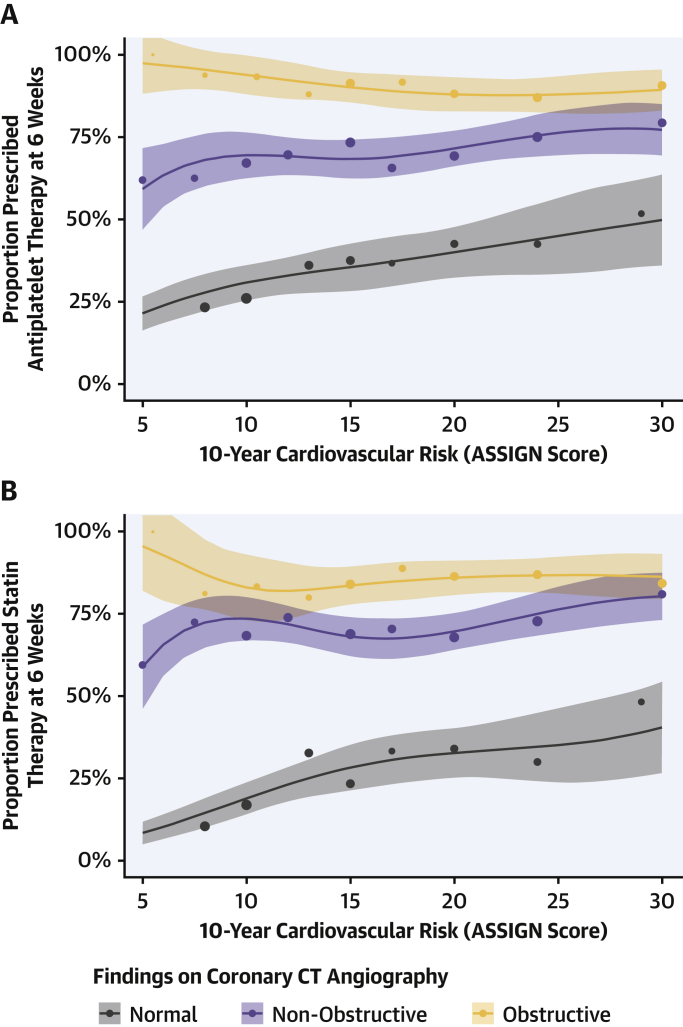
Interaction Between Coronary CT Angiography Findings and Clinically Estimated Cardiovascular Risk in Relation to Prescribing of Preventative Therapy Frequency of prescribing for **(A)** antiplatelet and **(B)** statin therapy at 6 weeks in patients with obstructive **(orange)** and nonobstructive **(purple)** coronary artery disease, and normal coronary arteries **(gray)** on coronary computed tomography (CT) angiography across a range of 10-year cardiovascular risk as determined from the ASSIGN score (11). The **lines and corresponding shaded areas** represent the prescribing estimates and 95% confidence intervals derived from a regression model. The **dots** represent the observed prescribing rates among the trial cohort grouped according to ASSIGN score with size proportional to the number of patients included in each group.

Within the coronary CTA group, 99% of participants with evidence of inducible ischemia on exercise testing were already receiving preventative medications at the baseline visit. In contrast, only 50% of participants were receiving preventative treatments in those who had a normal exercise test and evidence of nonobstructive or obstructive coronary artery disease on coronary CTA (Online Table 2). Following clinic consultation, 1 in 20 patients (5%) had their treatment altered at 6 weeks in the standard care arm compared with nearly 1 in 4 (23%) in the coronary CTA group (Table 4) (4). In particular, there were differences in the prescription of both antiplatelet and statin therapies that were sustained over the 5 years of follow-up (Figure 4). Of note, antiplatelet therapy use fell from 48% (baseline) to 41% (at 1 year) in the standard of care arm (p < 0.001), whereas it increased from 49% (baseline) to 52% (at 1 year) in those assigned to coronary CTA (p = 0.017). In contrast, overall statin use increased in both groups (standard care: 43% to 50%; coronary CTA: 44% to 59%; p < 0.001 for both groups) but this was greater in those assigned to coronary CTA (p < 0.001). Compared with those without coronary heart disease, rates of antiplatelet and statin therapies were markedly higher in patients who had coronary heart disease documented on the coronary CTA despite comparable 10-year cardiovascular risk scores (Figure 5). This was most apparent in participants with lower cardiovascular risk scores.

**Table 4:** Prescribed Therapy and Coronary Revascularization According to Coronary CTA Findings

Coronary CTA Result	Antiplatelet Therapy		Statin Therapy		Coronary Revascularization
	Baseline	New∗	Baseline	New∗	During First Year
Normal (n = 649)	227 (35.0)	1 (0.2)	158 (24.3)	2 (0.4)	0 (0.0)
Nonobstructive (n = 680)	334 (49.1)	148 (42.8)	326 (47.9)	160 (45.2)	27 (4.0)
Obstructive (n = 452)	343 (75.9)	62 (56.9)	327 (72.3)	56 (44.8)	197 (43.6)
Prognostically important CAD on coronary CTA (n = 178)†	148 (83.1)	16 (53.3)	142 (79.8)	14 (38.9)	98 (55.1)

Values are n (%).

Abbreviations as in Tables 1 and 3.

∗ Denominator excludes those receiving therapy at baseline.

† Prognostically important CAD defined as any of the following: ≥50% stenosis of left main coronary artery; ≥70% stenosis of at least 3 main epicardial arteries; or ≥70% stenosis of at least 2 epicardial arteries including the proximal left anterior descending artery.

### Modeling treatment effects

For the baseline model, there were 48.5 and 82.4 estimated events in the coronary CTA and standard care groups, respectively. This was similar to the observed number of events in each group (48 and 81 events, respectively). Under the counterfactual scenario where the proportion of participants receiving preventative therapy was the same in both arms, the estimated number of events in the coronary CTA group was 84.4 (i.e., similar to that observed in the control arm).

## Discussion

We report a post hoc analysis of the long-term follow-up of the SCOT-HEART trial participants in response to questions raised about the consistency and plausibility of the reported effect size. We demonstrate a consistency of effect on event rates across the differing trial subpopulations in keeping with their presentation and risk profile. We further demonstrate that greater diagnostic certainty led to better categorization and risk stratification with increased use of procedural and pharmacological interventions in the coronary CTA group. As a result of this improved diagnostic performance and risk classification, we can plausibly account for much of the observed effect size by the targeted application of these preventative interventions.

The SCOT-HEART trial included a broad range of patients who were at low, intermediate, and high risk of coronary events, as well as patients with prior coronary heart disease 7, 15. In the current analysis, we categorized patients according to the National Institute of Health and Care Excellence classification into those with nonanginal chest pain, possible angina, and known coronary heart disease (Central Illustration). Those participants with nonanginal chest pain had the lowest event rates, and these were linear over time. Moreover, the apparent benefits of coronary CTA were slow to accrue with a continuous rate of separation in events with time that appeared to take >1 year to be realized. These features are consistent with the event rates and time course of effects seen in primary prevention trials (16). In contrast, participants with possible angina had a higher and time-varying event rate with increased early hazards consistent with some individuals having higher risk disease, namely new onset angina 4, 17, 18. Patients with known coronary heart disease were already on established preventative therapies and the benefit appears to be rapid and early, consistent with an effect of coronary revascularization.

**Central Illustration:**
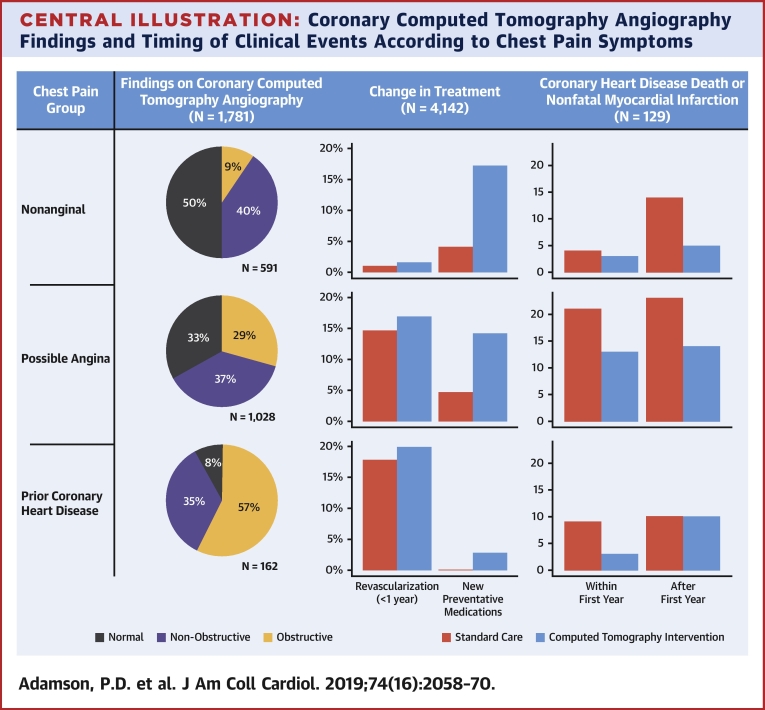
Coronary Computed Tomography Angiography Findings and Timing of Clinical Events According to Chest Pain Symptoms Findings on coronary computed tomography angiography **(left),** changes in provision of preventative medications and early coronary revascularization **(center),** and timing of coronary heart disease death or nonfatal myocardial infarction events **(right)** according to the National Institute of Health and Care Excellence guideline classification of chest pain symptoms.

One of the main advantages of coronary CTA is its negative predictive value. As such, it moves beyond traditional symptom assessment and ischemia testing, providing confidence to clinicians and patients alike regarding the absence of disease. This was reflected in the increased number of patients in whom a diagnosis of angina was excluded and preventive therapies discontinued in the coronary CTA group 3, 4. We observed that the event rate among this group was much lower than the equivalent diagnostic classification in the standard care group, which is consistent with the high negative predictive value and diagnostic accuracy of coronary CTA and implies greater diagnostic misclassification of patients in the standard care arm contributed to their higher event rate. False negative misclassification is common and previous reports (19) have highlighted that up to one-third of myocardial infarctions occur in patients who had previously been diagnosed with noncardiac chest pain. This finding was reproduced in the standard care group of the SCOT-HEART trial. Moreover, our modeling suggests that the use of coronary CTA can, via increased identification and treatment of previously unrecognized predominantly nonobstructive coronary heart disease, plausibly reduce event rates among those with nonanginal chest pain.

Coronary CTA is the only widely available and well validated noninvasive diagnostic technique able to identify nonobstructive coronary plaque disease. Standard approaches focusing on noninvasive ischemia testing have relatively poor sensitivity for obstructive coronary disease and, by definition, are unable to differentiate patients with normal coronary arteries from those with nonobstructive plaque disease. Furthermore, we have shown for the first time that the identification of nonobstructive coronary disease was a key factor associated with de novo provision of evidence-based preventative therapies among those who underwent coronary CTA.

Coronary revascularization was more common in the coronary CTA group during the first year after randomization. We here show this reflects increased early detection of obstructive coronary artery disease that includes left main stem and triple vessel disease. Coronary revascularization may be particularly impactful in those with prognostic disease and those with new onset, rapidly progressing, or recurrent angina, in whom underlying atherosclerotic disease process may be more active. This is potentially important as coronary revascularization is known to reduce ischemic coronary events and improve clinical outcomes in these more unstable clinical contexts 20, 21. In contrast, beyond 1 year, rates of coronary revascularization were higher in those who had received standard care alone and many of these revascularization episodes were triggered by myocardial infarction. This suggests that standard care may be associated with missed diagnoses of coronary artery disease that later declare themselves, whereas coronary CTA correctly identifies those who require treatment and thereby reduces future events.

There were important differences in the changes to pharmacological therapies seen within the 2 trial groups. First, antiplatelet therapy use fell in the standard care group but rose in the coronary CTA group, whereas statin use increased in both groups albeit there were greater increases in the coronary CTA group. Antiplatelet therapy at baseline in part reflects diagnostic uncertainty on the part of the primary care physician who may have initiated preventative therapy while awaiting specialist review. The high diagnostic sensitivity of coronary CTA resulted in an increased diagnosis of coronary artery disease (especially nonobstructive disease) and consequently increased prescribing of antiplatelet therapy in this group. In contrast, the reduced ability to detect nonobstructive coronary disease in the standard care group resulted in treatment discontinuation in many participants. Although the overall differences in prescribing may appear modest, it should not be forgotten, it is the distribution of such therapies that is important and many patients in the standard care group may be receiving futile treatments because many will not have underlying coronary disease. For the first time, we here report that those undergoing coronary CTA will have more directed and appropriate therapies. Indeed, when we look at those who underwent coronary CTA, the rates of antiplatelet and statin therapy use were almost 3-fold higher in those with coronary artery disease compared with those without disease despite identical 10-year cardiovascular risk scores. Thus, simply looking at the overall frequency of prescribing of preventative therapies ignores the importance of how such medications are distributed within a population. Again, these findings suggest that coronary CTA is consistent with a precision medicine approach by identifying the most appropriate treatment for each patient.

Using treatment effect estimates from published randomized controlled trials, systematic reviews, and meta-analyses 14, 16, 21, 22, 23, 24, 25, 26, we explored for the first time whether the effect of additional treatment with antiplatelet, statin, and revascularization interventions could account for the observed effect size. Whereas this modeling required a number of assumptions, they were nonetheless reasonable, and more reasonable than assuming that patients receiving additional treatment in the coronary CTA group shared the same cardiovascular event rate as the entire cohort. This modeling allowed us to demonstrate that at least some of the effect size we observed was plausible and could be accounted for by current evidence. In addition, we would also highlight that the effect size reported in the SCOT-HEART trial is consistent with rates of myocardial infarction reported in other trials of coronary CTA in patients with stable chest pain 5, 27 and meta-analyses 28, 29, as well as large-scale observation studies (30). Moreover, our model neither accounts for positive lifestyle changes, such as smoking cessation, nor treatment compliance, which may also have a beneficial impact given the greater uptake of antiplatelet and statin therapy in those diagnosed with coronary artery disease by coronary CTA. We accept that many of our modeling choices are necessarily subjective, and we are pleased to provide access to the data to allow other researchers to test the effect of different assumptions. Finally, we would highlight that the magnitude of the effect size we described in the SCOT-HEART trial is consistent with other diagnostic studies in patients with suspected coronary heart disease (14), as well as primary prevention trials in patients with interventions targeted to those at higher risk, such as the JUPITER (Justification for the Use of Statins in Primary Prevention: An Intervention Trial Evaluating Rosuvastatin) trial (20).

The identification of disease is inextricably linked to downstream changes in lifestyle, initiation and intensification of preventative therapies, and the judicious use of coronary revascularization. The combined effect of all of these interventions on coronary atherosclerosis reduces the future risk of myocardial infarction. In this regard, it was intriguing to observe that the benefits of coronary CTA were similar regardless of presentation symptoms and indeed the greatest proportionate benefit was seen in patients without a diagnosis of angina due to coronary heart disease. This begs the question of whether coronary CTA has a role in primary prevention of coronary heart disease, and this will be the subject of the forthcoming SCOT-HEART 2 trial.

### Study limitations

Some have highlighted that there may have been bias in the reporting of outcomes in the SCOT-HEART trial: something that should always be considered in an open-label trial. However, we think this is unlikely. First, the delayed separation of the event curves suggests that there was no early bias in event reporting because the CT result would be available after 2 weeks, whereas treatment changes took a further 4 to 6 weeks to implement. Second, the finding that coronary CTA was associated with less normal invasive coronary angiography and higher early rates of revascularization suggests that the coronary CTA more accurately identified the disease process. Third, coronary CTA increased the rate of diagnosis of coronary artery disease and intuitively would be more inclined to increase the diagnosis of myocardial infarction, which would act against any potential benefits of the trial intervention. Fourth, the early increase followed by later reductions in coronary revascularization in the coronary CTA group does not support the postulation of ascertainment bias. Why would these rates suddenly change direction? Fifth, the patterns of event reduction are consistent across the trial subpopulations with both lower risk nonanginal chest pain and higher risk new onset angina pectoris responding to changes in treatment as previously reported in primary and secondary prevention trials. Sixth, although we did not undertake clinical endpoint adjudication, we do not believe that this is likely to have affected our findings. A large analysis involving 10 cardiovascular trials has demonstrated the process of blinded endpoint adjudication changes neither the number of endpoint events identified nor the overall treatment effect estimate for the randomized intervention (31). A recent Cochrane Systematic Review (32) has also reported similar trial outcomes irrespective of central versus local endpoint determination. Ultimately, for a pragmatic trial determining the effect of introducing a new diagnostic test into a health care system, the main goal is to establish the impact on health care outcomes as reported by that health care system. We believe clinical outcomes reported by routine health records data remain the most appropriate, independent, and accurate method of clinical endpoint ascertainment for the SCOT-HEART trial. Finally, we acknowledge our modeling approach does assume that the highest risk patients were those who had received additional preventative therapies as a result of the coronary CTA intervention, and this may have overestimated some of the benefits. This is particularly applicable to the assumption of treatment benefit derived from coronary revascularization. Although we chose treatment effects applicable to an unstable angina population, we believe this is justifiable as the shape of the instantaneous hazard curves supports a high short-term risk that plateaus after 6 to 12 months in a pattern consistent with acute coronary syndrome populations.

## Conclusions

We have presented a multifaceted analysis that consistently and robustly demonstrates the plausibility of a reduction in long-term coronary events consequent on investigating patients with stable chest pain using coronary CTA. If we are to improve the prevention of future myocardial infarction, coronary CTA would appear to be the most effective and indeed the only proven investigative approach in patients with stable chest pain.

Perspectives **COMPETENCY IN PATIENT CARE AND PROCEDURAL SKILLS:** Adding coronary CTA to standard care in patients with stable chest pain syndromes can reduce myocardial infarction and death over 5 years by facilitating evidenced therapy for those with coronary artery disease independent of symptom characteristics.

**TRANSLATIONAL OUTLOOK:** Future studies should evaluate the value of coronary CTA to guide treatment of asymptomatic patients at risk of coronary events.
