# Supplementary material for: Guiding Therapy by Coronary CT Angiography Improves Outcomes in Patients With Stable Chest Pain
Source: J Am Coll Cardiol. 2019 Oct 22;74(16):2058–70. doi: 10.1016/j.jacc.2019.07.085 (PMC6899446; doi:10.1016/j.jacc.2019.07.085)
Supplement: Online Data [file mmc1.docx]

**SUPPLEMENTARY APPENDIX**

##### Guiding Therapy by Coronary CT Angiography Improves Outcomes in Patients with Stable Chest Pain

Philip D. Adamson MD, PhD,^1,2^ Michelle C. Williams MD, PhD,^1,3^ Marc R. Dweck, MD, PhD,^1,3^ Nicholas L. Mills, MD, PhD,^1,3^ Nicholas A. Boon MD,^1^ Marwa Daghem MD,^1,3^ Rong Bing MD,^1,3^ Alastair J. Moss MD,^1,3^ Kenneth Mangion MD, PhD,^4^ Marcus Flather MD, PhD,^5^ John Forbes MD, PhD,^6^ Amanda Hunter MD,^1,3^ John Norrie PhD,^7^ Anoop S.V. Shah MD, PhD,^1,3^ Adam D. Timmis MD, PhD,^8^ Edwin J.R. van Beek MD, PhD,^3^ Amir A. Ahmadi MD,^9,10^ Jonathon Leipsic MD,^10^ Jagat Narula MD, PhD,^9^ David E. Newby MD DSc,^1,3^ Giles Roditi MD PhD,^4^ David A. McAllister MD MPH*,^9^ Colin Berry MD PhD*,^4^on behalf of The SCOT-HEART Investigators

### *Equal contribution

### **Affiliations**

^1^British Heart Foundation Centre for Cardiovascular Science, University of Edinburgh, Edinburgh, UK

^2^Christchurch Heart Institute, University of Otago, Christchurch, New Zealand,

^3^Edinburgh Imaging, Queen’s Medical Research Institute University of Edinburgh, Edinburgh, UK

^4^Institute of Cardiovascular and Medical Sciences, University of Glasgow, Glasgow, UK

^5^Norwich Medical School, University of East Anglia, Norwich, UK

^6^Health Research Institute, University of Limerick, Limerick, Ireland

^7^Edinburgh Clinical Trials Unit, University of Edinburgh, Edinburgh, UK

^8^William Harvey Research Institute, Queen Mary University of London, London, UK

^9^Ichan School of Medicine and Mount Sinai Hospital, Mount Sinai Heart, New York, USA

^10^St Paul’s Hospital, University of British Columbia, Vancouver, Canada

^11^Institute of Health and Wellbeing, University of Glasgow, Glasgow, UK

**Address for correspondence:**

Dr Philip D. Adamson

Christchurch Heart Institute

University of Otago Christchurch

PO Box 4345

Christchurch

8140

New Zealand

Email: philip.adamson@cdhb.health.nz

**Methods for determining primary endpoint events (Coronary heart disease death or non-fatal myocardial infarction)**

Death due to CHD was identified where Scotland’s National Mortality Records recorded the primary cause of death with the following ICD-10 codes: I21, I22, I249, I462, or I256. Nonfatal myocardial infarction was determined using the same ICD-10 codes when these were listed within the first 6 discharge diagnoses.

**Methods for estimating number of primary events in the CT coronary angiography group in the counterfactual model**

To explore the plausibility of the treatment effect, we performed a modelling study aiming to estimate the mean outcome for patients in the coronary CT angiography group under the following counterfactual scenario: wherein the management of patients in the coronary CT angiography group was the same as that observed in the standard care group. We compared the observed event rate in the coronary CT angiography group to a simulated event rate under the following counterfactual scenario: the management of patients in the coronary CT angiography group was the same as that observed in the standard care group (1). We first calculated the proportion of participants in each group who were prescribed antiplatelet and statin therapy at 6 weeks and 1 year as well as the number of coronary revascularization procedures performed in the first year after randomization. For each intervention, we then obtained treatment effect estimates from the published literature for the likely efficacy of each outcome (2–8). Based on our review, we assigned the following effect estimates: antiplatelet therapy prescribed during the first year = 0.5, antiplatelet therapy prescribed subsequent years = 0.7; statin therapy prescribed at any time point = 0.7; coronary revascularization performed during the first year = 0.7, coronary revascularization performed subsequently = 1.0. We then constructed a “baseline” model for the primary outcome in the coronary CT angiography group using a Cox regression model. We subsequently applied the treatment effect estimates to the baseline model, to model the effect on the primary outcomes of the additional therapy received in the coronary CT angiography group.

The modelling process proceeded in 7 steps:

| **Step 1** | We reviewed the published literature regarding the beneficial effects for selected cardiovascular therapies (antiplatelet and statin therapies and coronary revascularisation) in the setting of primary and secondary prevention in order to identify the risk/hazard ratios (RR/HR) for myocardial infarction within the treatment arms of these trials. The corresponding RR were:   - Aspirin: 0.45 (95% CI: 0.35 to 0.58) in unstable angina (3,9) - Aspirin: 0.69 (95% CI: 0.60 to 0.80) in stable coronary disease (6) - Statins: 0.69 (95% CI: 0.65 to 0.73) in primary or secondary prevention (7) - Coronary revascularisation: 0.79 (95% CI: 0.63 to 1.00) with some variation according to duration of follow-up (2)   - Early (<6 months) myocardial infarction: 0.69 (95% CI: 0.43 to 1.08)   - Intermediate (6–12 months) myocardial infarction: 0.79 (95% CI: 0.63 to 1.00)   - Late (>12 months) myocardial infarction: 0.78 (95% CI: 0.67 to 0.92) |
| --- | --- |
| **Step 2** | Accordingly, we assigned the following RR to the counterfactual analysis.   - Aspirin during first year after randomization: 0.5 - Aspirin in subsequent years: 0.7 - Statins (any time point): 0.7 - Coronary revascularization (performed during first year): 0.7 |
| **Step 3** | We determined the number of additional patients who received each of these treatments in the CT coronary angiography group compared with the standard care group. |
| \|  \|  \| Treatment received \|  \| \| --- \| --- \| --- \| --- \| \|  \|  \| During first year post-randomization \| Subsequent years \| \| Antiplatelet therapy \| Standard care \| 1006 (49%) \| 842 (41%) \| \|  \| CT coronary angiography \| 1192 (58%) \| 1,083 (52%) \| \|  \| Net difference \| 186 \| 241 \| \| Statin therapy \| Standard care \| 952 (46%) \| 1,038 (50%) \| \|  \| CT coronary angiography \| 1092 (53%) \| 1,229 (59%) \| \|  \| Net difference \| 140 \| 191 \| \| Coronary revascularization \| Standard care \| 208 \| 59 \| \|  \| CT coronary angiography \| 246 \| 12 \| \|  \| Net difference \| 38 \| −47 \| | |
| **Step 4** | Using a “baseline” adjusted Cox proportional hazards model with estimated the risk of a primary endpoint event for each individual. The covariates included in this model included:   - Randomised treatment allocation - Age - Sex - Total and HDL cholesterol concentration - Body mass index - Diabetes mellitus - Prior coronary heart disease - Atrial fibrillation - Chest pain typicality - Results of the exercise electrocardiogram - Systolic and diastolic blood pressure - Smoking status - Prior cerebrovascular or peripheral vascular disease - 10-year estimated cardiovascular risk (ASSIGN score) - Baseline and 6 week medications (antiplatelet, statins, renin-angiotensin system inhibitors, beta-blockers - Baseline clinical diagnosis |
| **Step 5** | Using the linear predictor (LP) from this model, we ranked patients in the CT coronary angiography group according to their predicted risk of an event, in descending order. For each treatment, dummy variables were then created whereby the N highest-risk individuals observed to receive a treatment were assigned a value of 1, with all other individuals assigned a value of zero, where 1 = not treated under the counterfactual and 0 = treated as per observed under the counterfactual. N for each treatment was set to the number of *additional* patients started on that treatment in the CT arm compared to the control arm. |
| **Step 6** | We then estimated the event rate under the observed circumstances, and under the counterfactual using the survest function in the rms package. These were calculated as follows:  **Modelled survival probability under treatment as observed**  $LP_{obs}=\boldsymbol{\beta}*\boldsymbol{X}$  $S_{obs}(5)=S_{0}(5)^{e^{LP_{obs}}}$  **Modelled risk under treatment as per counterfactual**  $LP_{cnt}=LP_{obs}+aspirin\_year\_one*log\left( \frac{1}{0.5} \right)+aspirin\_other*log\left( \frac{1}{0.7} \right)+statin*log\left( \frac{1}{0.7} \right)+ revascularisation*log\left( \frac{1}{0.7} \right)$  $S_{cnt}(5)=S_{0}(5)^{e^{LP_{cnt}}}$  LP is linear predictor, X and beta are the design matrix and vector of coefficients corresponding to the model described in step 5, S(5) is the survival probability at the end of year 5, with subscripts 0, cnt and obs referring to the survival at the mean of all covariates, the survival under the observed treatments, and the survival under the counterfactual treatments respectively. The antiplatelet, statin, and PCI variables are the dummy variables referred to in step 5. Note that the baseline survival probabilities are exponentiated by (e^LP^), not multiplied. |
| **Step 7** | Next, the observed $S_{obs}(5)$ and counterfactual $S_{cnt}(5)$ survival probabilities were each summed for all participants in the CT arm to estimate the total number of events under the observed and counterfactual scenarios. |

**
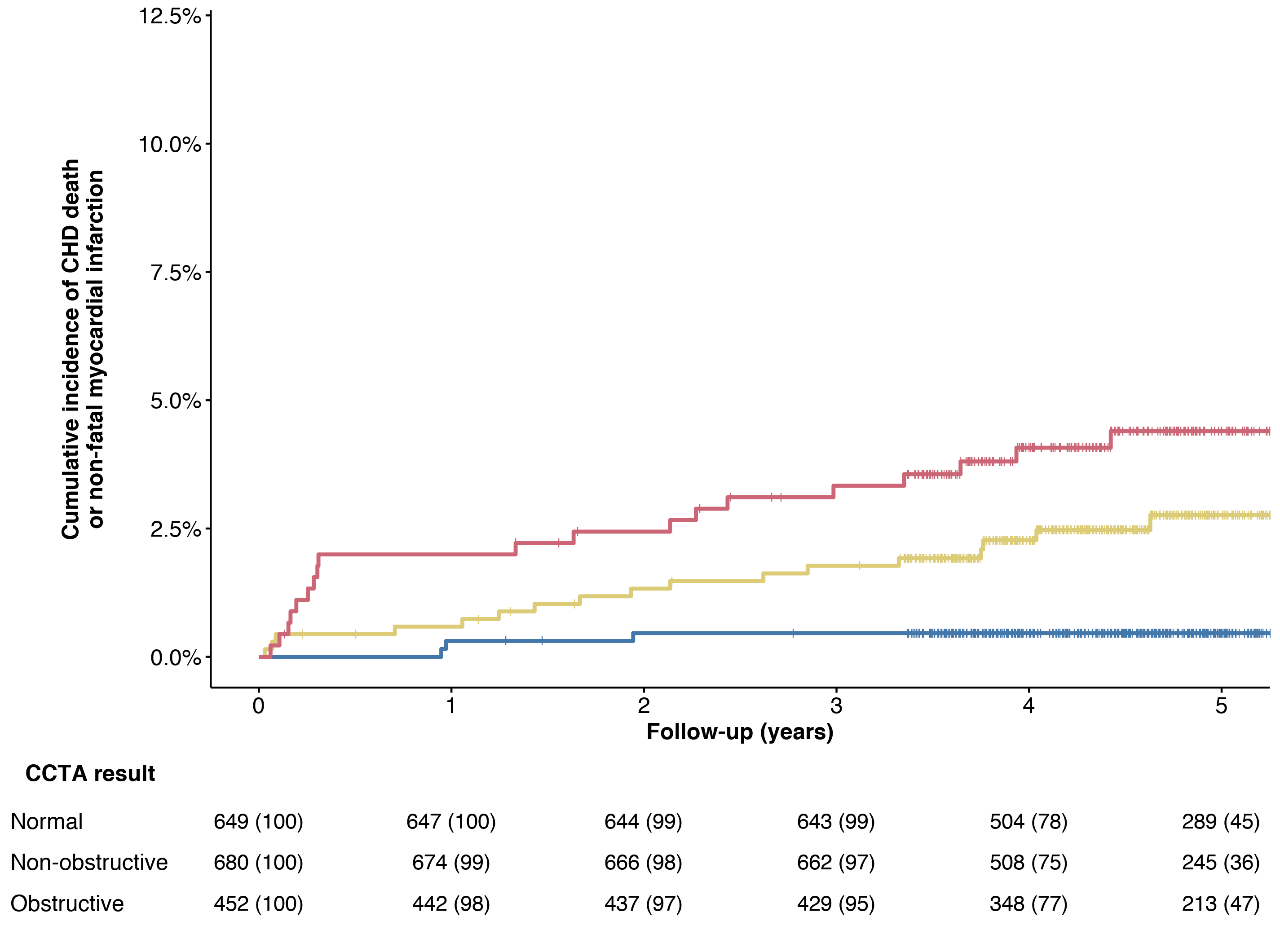
**

**Supplementary Figure 1**. **Cumulative incidence of the primary endpoint according to coronary CT angiography findings**.

Cumulative incidence curves for coronary heart disease death or non-fatal myocardial infarction in patients with normal coronary arteries (blue), non-obstructive coronary disease (yellow), and obstructive coronary disease (red) demonstrating the clear gradient of risk associated with these findings.

CHD, coronary heart disease; CCTA, coronary computed tomography angiography.

**Supplementary Tables**

**Supplementary Table 1: Findings on invasive coronary angiography performed within 1 year of randomization according to the presence or absence of inducible ischemia of exercise ECG.**

|  | Findings on exercise ECG test | | | | | |
| --- | --- | --- | --- | --- | --- | --- |
|  | Normal | | Inconclusive | | Abnormal | |
|  | Standard care | CCTA | Standard care | CCTA | Standard care | CCTA |
| Number of diseased vessels* |  |  |  |  |  |  |
| 0 | 46 (68.7) | 49 (53.3) | 39 (60.0) | 31 (40.8) | 45 (26.5) | 44 (26.7) |
| 1 | 17 (25.4) | 28 (30.4) | 14 (21.5) | 25 (32.9) | 59 (34.7) | 59 (35.8) |
| 2 | 3 (4.5) | 8 (8.7) | 9 (13.8) | 11 (14.5) | 29 (17.1) | 37 (22.4) |
| 3 | 1 (1.5) | 7 (7.6) | 3 (4.6) | 9 (11.8) | 37 (21.8) | 25 (15.2) |
| Prognostically important CAD^ | 2 (3.0) | 11 (12.0) | 5 (7.7) | 13 (17.1) | 57 (33.5) | 48 (29.1) |

*Diseased vessels defined as ≥70% stenosis of any main epicardial artery or ≥50% stenosis of the left main coronary artery

^Prognostically important CAD defined as any of the following: ≥50% stenosis of left main coronary artery; or ≥70% stenosis of at least 3 main epicardial arteries; or ≥70% stenosis of at least 2 epicardial arteries including the proximal left anterior descending artery.

ECG, electrocardiogram; CCTA, coronary computed tomography angiography; CAD, coronary artery disease

**Supplementary Table 2: Baseline prescription for preventative therapies according to the presence or absence of inducible ischemia on exercise ECG and the severity of coronary disease**

|  | Findings on exercise ECG test | | | | | |
| --- | --- | --- | --- | --- | --- | --- |
|  | Normal | | Inconclusive | | Abnormal | |
| Findings on CCTA | n | Receiving preventative therapy at baseline | n | Receiving preventative therapy at baseline | n | Receiving preventative therapy at baseline |
| Normal | 409 | 132 (32.3) | 79 | 56 (70.9) | 31 | 29 (93.5) |
| Mild plaque (<50% stenosis) | 224 | 92 (41.1) | 60 | 49 (81.7) | 29 | 28 (96.6) |
| Moderate plaque (50%–70% stenosis) | 155 | 78 (50.3) | 42 | 37 (88.1) | 40 | 40 (100.0) |
| Obstructive plaque (≥70% stenosis) | 139 | 91 (65.5) | 76 | 66 (86.8) | 136 | 136 (100.0) |

ECG, electrocardiogram; CCTA, coronary computed tomography angiography.

**Supplementary References**

1. Rubin DB. The design versus the analysis of observational studies for causal effects: parallels with the design of randomized trials. Stat Med 2007;26:20–36.

2. Fanning JP, Nyong J, Scott IA, Aroney CN, Walters DL. Routine invasive strategies versus selective invasive strategies for unstable angina and non-ST elevation myocardial infarction in the stent era. Cochrane Database Syst Rev 2016:Cd004815.

3. Verheugt FWA. Acute coronary syndromes: Drug treatments. The Lancet 1999;353:s20–s23.

4. Theroux P, Ouimet H, McCans J et al. Aspirin, heparin, or both to treat acute unstable angina. N Engl J Med 1988;319:1105–11.

5. Ridker PM, Danielson E, Fonseca FA et al. Rosuvastatin to prevent vascular events in men and women with elevated C-reactive protein. N Engl J Med 2008;359:2195–207.

6. Antithrombotic Trialists’ Collaboration. Aspirin in the primary and secondary prevention of vascular disease: collaborative meta-analysis of individual participant data from randomised trials. Lancet 2009;373:1849–1860.

7. National Institute for Health and Care Excellence. Lipid modification: cardiovascular risk assessment and the modification of blood lipids for the primary and secondary prevention of cardiovascular disease. Clinical Guideline 181. London: NICE, 2014.

8. Boden WE, O'Rourke RA, Teo KK et al. Optimal Medical Therapy with or without PCI for Stable Coronary Disease. N Engl J Med 2007;356:1503–1516.

9. Antiplatelet Trialists' Collaboration. Collaborative overview of randomised trials of antiplatelet therapy—I: Prevention of death, myocardial infarction, and stroke by prolonged antiplatelet therapy in various categories of patients. BMJ 1994;308:81–106.
